# Low-Density Granulocytes Affect T-SPOT.TB Assay by Inhibiting the Production of Interferon-γ in T Cells via PD-L1/PD-1 Pathway

**DOI:** 10.3389/fmicb.2020.622389

**Published:** 2021-01-28

**Authors:** Jiayue Rao, Rigu Su, Yiping Peng, Yang Guo, Zikun Huang, Yutao Ye, Yujie Gao, Jun Liu, Lu Zhang, Qing Luo, Junming Li

**Affiliations:** ^1^Department of Clinical Laboratory, The First Affiliated Hospital of Nanchang University, Nanchang, China; ^2^Department of Tuberculosis, Jiangxi Chest Hospital, Nanchang, China

**Keywords:** low-density granulocytes, T-SPOT.TB, programmed death ligand 1, tuberculosis, T cells

## Abstract

**Background:**

T-SPOT TB (T-SPOT) assay is widely used for detection of *Mycobacterium tuberculosis* infection that is based on the detection of *M. tuberculosis*-specific interferon-γ-secreting T cells (ISCs) in peripheral blood mononuclear cells (PBMCs). Recently, high frequencies of low-density granulocytes (LDGs) were found in the PBMCs of tuberculosis patients. Whether these LDGs affect the detection of T-SPOT has not been investigated. The impact of LDGs on T-SPOT assay and related mechanism were investigated in this study.

**Methods:**

The correlations between the frequencies of LDGs and the results of T-SPOT were analyzed. T-SPOT with LDG-removed PBMCs and PBMCs with exogenous addition of LDGs were performed. The possible mechanism was explored by detecting the levels of negative immune regulatory molecules on LDGs. The impact of programmed death ligand 1 (PD-L1) on T-SPOT was evaluated and confirmed by function blocking with neutralizing antibody.

**Results:**

The positive rates of T-SPOT and ISCs in tuberculosis patients with low LDGs frequency (*n* = 22) were significantly higher than those with high LDGs frequency (*n* = 39). Removal or exogenous addition of LDGs significantly increased or decreased the ISCs and the positive rate of T-SPOT. The frequencies of interferon-γ-producing T cells were negatively correlated with the frequencies of LDGs. The expression of PD-L1 was significantly elevated on LDGs. Pretreatment of LDGs with anti-PD-L1 antibody significantly counteracted the impact of LDGs on T-SPOT. Treatment of PBMCs with anti-PD-L1 antibody resulted in comparable ISCs with that of LDG removal.

**Conclusion:**

LDGs can inhibit the production of interferon-γ in T cells and decrease the positive rated of T-SPOT assay via highly expressed PD-L1.

## Introduction

Tuberculosis is one of the top 10 causes of death worldwide and the leading cause of death from a single infectious agent. As reported in the Global Tuberculosis Report 2019 (World Health Organization [WHO], 2019), approximately 1.7 billion people were estimated to be infected with *Mycobacterium tuberculosis* (*Mtb*) in a latent infection state, of which 5–10% likely progress to active tuberculosis in their life. However, the risk of reactivation of latent tuberculosis is remarkably elevated among immunocompromised individuals, such as individual with autoimmune diseases, cancer, diabetics, or HIV infection (Balbi et al., 2018; Ho and Leung, 2018; Lee et al., 2018). Therefore, it is very important to find these infected individuals out in populations with high risk of tuberculosis activation. On the other hand, due to the lack of sensitive and specific diagnosis methods, the diagnosis of tuberculosis still faces great challenges, especially for the patients with negative bacteriological detection (Singhania et al., 2018).

T-SPOT.TB (T-SPOT) assay is the most commonly used method for screening or ruling out the infection of *Mtb* worldwide (World Health Organization [WHO], 2018; Haas and Belknap, 2019), which has been included in the guidelines of tuberculosis diagnosis and treatment in more than 20 countries. The first and key step of T-SPOT assay is to separate mononuclear cells from peripheral blood by Ficoll density gradient centrifugation, followed by culturing and stimulating these peripheral blood mononuclear cells (PBMCs) with *Mtb* antigens to induce the interferon-gamma (IFN-γ) secretion in *Mtb*-specific T cells, which had been sensitized by *Mtb* antigens in infected human body. Finally, the amount of IFN-γ secreting T cells (ISCs) was detected by ELISPOT technique. Therefore, for T-SPOT assay, obtaining high-quality PBMCs is the premise of obtaining reliable detection results.

However, recent researches found that the PBMCs obtained by density gradient centrifugation are often mixed with a population of neutrophils in tuberculosis patients and some other patients with high risk of tuberculosis activation, such as patients with autoimmune disease, cancer, or HIV infection (Denny et al., 2010; Cloke et al., 2012; Sagiv et al., 2015; Deng et al., 2016). These granulocytes are called low-density granulocytes (LDGs) because of their lower density than those of normal-density granulocytes (NDGs).

In this study, we investigated whether these abnormally elevated LDG in PBMCs affect T-SPOT assay and the related mechanism. Our results show that LDGs can significantly downregulate the positive rate of T-SPOT test, and the main mechanism is related to the high expression of programmed death ligand 1 (PD-L1) on LDGs, which can downregulate the activation of T cells and the production of IFN-γ in T cells.

## Materials and Methods

### Subjects and Samples

A total of 186 patients with pulmonary tuberculosis were recruited from the Jiangxi Chest Hospital from November 2018 to October 2019. The diagnostic criteria for pulmonary tuberculosis were in accordance with the “Guideline of Clinical Diagnosis and Treatment: Tuberculosis Section of China” (Chinese Medical Association, 2005). All patients in this study were confirmed by bacteriological test, including smear microscopy or *Mtb* culture. Fourteen age-and sex-matched, T-SPOT-negative asymptomatic healthy volunteers were recruited in the same period. All subjects with pregnancy, autoimmune diseases, cancer, HIV infection, diabetes mellitus, chronic renal failure, chronic liver disease, and current or recent infection were excluded from the study.

This study was approved by the Ethical Review Committees (number: 2018YYLS006) of The First Affiliated Hospital of Nanchang University and was carried out in compliance with the Helsinki Declaration. Written informed consent was obtained from all the subjects enrolled in this study. All experiments are carried out in the biosafety level 2 laboratory.

### Cell Isolation

The PBMCs and NDGs fractions were isolated from the peripheral blood samples using density gradient centrifugation as described previously (Su et al., 2019). LDGs were purified from the PBMC fraction by immune-magnetic negative isolation using the EasySep Direct Human Neutrophil Isolation Kit (STEMCELL Technologies, Vancouver, Canada) in accordance with the manufacturer’s instructions. The LDGs were identified as CD14^*low*^ CD15^+^ granulocytes by flow cytometry (FCM).

The removal of LDGs from PBMCs was performed by immune-magnetic positive isolation using the EasySep Human CD15 Positive Selection Kit (STEMCELL Technologies, Vancouver, Canada) according to the manufacturer’s instructions. The viability of cells was determined by Trypan blue staining, and samples with more than 95% viable cells were used in subsequent experiments.

### Bacterial Strains and Infection

*Mtb* H37Rv strain was cultured at 37°C up to early mid-log phase in Middlebrook 7H9 broth, then killed at 95°C for 30 min. To obtain single-cell suspension, the bacterial aggregates were treated with an ultrasonic shaker for 5 min, left standing for 15 min, then collected and density adjusted to 0.5 at OD600 (approximately 10^7^ bacteria/ml). For *in vitro* infection, whole blood was infected with *Mtb* at the multiplicity of infection (MOI) of 5 (bacteria to neutrophils) as described previously (Su et al., 2019).

### Flow Cytometry Analysis

Cells were incubated with FcR blocking reagent for 5 min followed by incubating with or without the mixture of fluorescent-labeled antihuman monoclonal antibodies: CD14-ECD, CD15-PE-Cy5, CD3-fluorescein isothiocyanate (FITC), and IFN-γ-PE (Beckman Coulter, Brea, CA, United States) for 30 min. For intracellular staining, cells were treated with IntraPrep Permeabilization Reagent (Beckman Coulter, Brea, CA, United States) before the staining. The Annexin V-FITC and propidium iodide were used to eliminate the dead cells from the analysis. Auto-fluorescence and non-specific staining were determined by using isotype-matched controls. The analysis was performed using a Cytomics FC 500 flow cytometer (Beckman Coulter, Brea, CA, United States).

### T-SPOT.TB Assays

The T-SPOT.TB test (Oxford Immunotec Ltd., Abingdon, United Kingdom) was performed according to manufacturer’s instructions. Peripheral venous blood was collected in vacuum containers containing heparin anticoagulant and processed within 4 h of phlebotomy. The PBMCs fractions were isolated by Ficoll density gradient centrifugation, cultured in microtiter plate wells that were precoated with anti-IFN-γ antibodies at 1.5 × 10^5^ cells per well, in the presence of ESAT-6 and CFP-10, or phytohemagglutinin (PHA) as a positive control, or equal volume of AIM-V medium as a negative control. After incubation for 18–20 h, alkaline phosphatase-conjugated secondary antibody and chromogenic substrate were added to form visible spots. Spot counting was performed by vSPOT ELISPOT reader (Autoimmun Diagnostika GmbH, Germany). Results are expressed as spot-forming cells or IFN-γ-secreting cells (ISCs). Result is judged as positive when the number of spots in any test well (ESAT-6 and CFP-10 stimulation wells) minus the number of spots in the negative control well is ≥6, or when the number of spots in any test well is ≥2 times of the number of spots in the negative control well.

### Statistical Analysis

Values were presented as mean ± *SD* or mean ± standard error of the mean (SEM) and analyzed using GraphPad Prism 5.01 software. Kolmogorov–Smirnov test was used to examine normal distribution. Independent samples *t*-test and one-way analysis of variance (ANOVA) were used when normal data distribution was confirmed, and Mann–Whitney U-test was used for the variables without normal distribution. Chi-square test was used for the comparison of frequencies between groups. Differences were considered statistically significant at *P* < 0.05.

## Results

### The Frequencies of LDGs Correlate With the Results of T-SPOT Detection in Tuberculosis Patients

**FIGURE 1:**
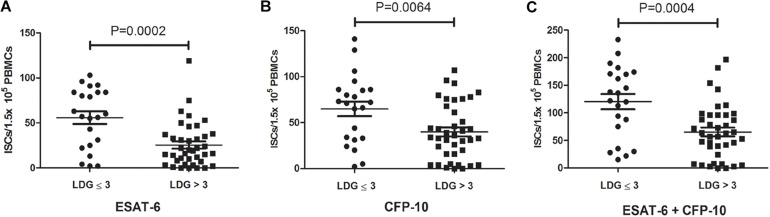
The numbers of interferon-γ-secreting cells (ISCs) in tuberculosis patients with high low-density granulocytes (LDGs) frequencies (>3%) are lower than that of low LDGs frequencies (≤3%). The numbers of ISCs response to **(A)** ESAT-6 and **(B)** CFP-10 in patients with high LDGs frequencies (*n* = 39) are significantly lower than those of low LDGs frequencies (*n* = 22). **(C)** The number of total ISCs in response to ESAT-6 and CFP-10 in patients with high LDGs frequencies is significantly lower than those of low LDGs frequencies. Each symbol denotes a single subject, and the mean ± *SD* for each study population is shown. Statistical significance was determined by an unpaired Student’s *t*-test.

According to the frequencies of LDGs in PBMCs, 61 tuberculosis patients were divided into two groups, high LDGs frequency group (LDGs > 3%, *n* = 39) and low LDGs frequency group (LDGs ≤ 3%, *n* = 22), and the positive rates of T-SPOT and the number of ISCs in these two groups were compared. Results showed that the positive rate of T-SPOT assay in high LDGs frequency group was significantly lower than that of low LDGs frequency group (Table 1). Furthermore, the number of ISCs in response to both ESAT-6 and CFP-10 stimulation was significantly lower in high LDGs frequency group than those of low LDGs frequency group (Figure 1).

**TABLE 1:** Results of T-SPOT assay in tuberculosis patients with high or low frequency of low-density granulocytes (LDGs).

LDGs frequency (%)	Negative	Positive	Positive rate (%)	*P*-values
≤3	0	22	100.00%	0.015
>3	9	30	76.92%	

### LDGs Do Not Secret IFN-γ in Response to Stimulation of *Mtb* Antigens

Considering that there is a high proportion of LDGs in the PBMCs of patients with tuberculosis and patients at high risk of tuberculosis reactivation, the first thing we want to know is whether these LDGs can also secrete IFN-γ under the stimulation of *Mtb* antigens.

**FIGURE 2:**
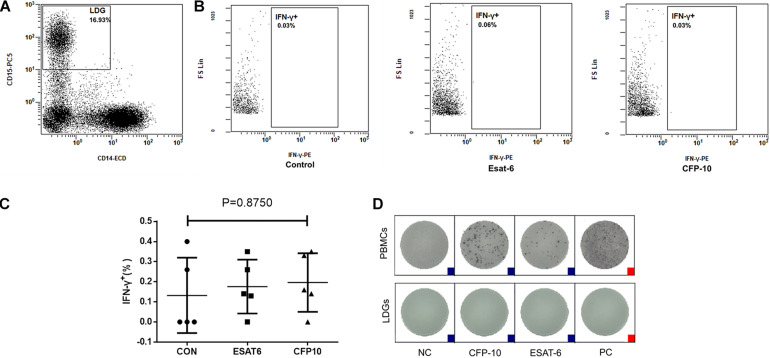
Detection of interferon-gamma (IFN-γ) production in low-density granulocytes (LDGs). Peripheral blood mononuclear cells (PBMCs) isolated from tuberculosis patients (*n* = 5) were stimulated with or without ESAT-6 or CFP-10 for 18–20 h. **(A)** LDGs were gated in PBMCs as CD14^*low*^ CD15^+^ populations by flow cytometry, and the intracellular IFN-γ of LDGs was detected by FCM. **(B)** Representative flow cytometry (FCM) detection images of a representative subject and the levels of intracellular IFN-γ were expressed as mean ± *SD*. **(C)** Statistical significance was determined by one-way analysis of variance (ANOVA). PBMCs or purified LDGs were used to T-SPOT assay, and **(D)** a group of result images of a single subject was shown as a representative result from 1/12 patients.

The PBMCs were isolated from tuberculosis patients and stimulated with ESAT-6 and CFP-10, respectively, followed by incubation for 18–20 h. The intracellular IFN-γ of LDGs was detected by FCM. Results showed that no matter stimulated or not, LDGs did not produce IFN-γ (Figures 2A–C). Furthermore, purified LDGs with purity of above 90% were detected for the secretion of IFN-γ by using T-SPOT.TB kit according to the manufacturer’s instruction, except that the PBMCs were replaced by purified LDGs. Results confirmed that LDGs do not secrete IFN-γ under the stimulation of *Mtb* antigens and PHA (Figure 2D).

### Removal of LDGs From PBMCs Increased Interferon-γ-Secreting Cells and the Positive Results of T-SPOT.TB Assay

**FIGURE 3:**
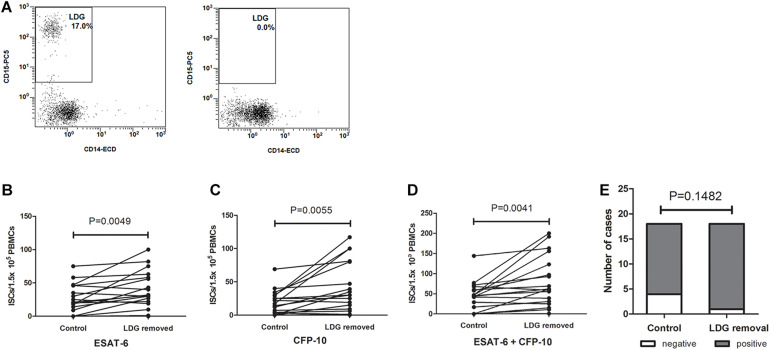
The numbers of interferon-γ-secreting T cells (ISCs) were increased after the removal of low-density granulocytes (LDGs). **(A)** The LDGs in peripheral blood mononuclear cells (PBMCs) of tuberculosis patients (*n* = 16) were removed by immunomagnetic separation, and T-SPOT assays were performed with the untreated PBMCs and LDG-removed PBMCs, respectively, with the same numbers of mononuclear cells. The ISCs in response to **(B)** ESAT-6 and **(C)** CFP-10 stimulation, and **(D)** the sum of the ISCs in response to ESAT-6 and CFP-10 were counted and compared by paired Student’s *t*-test. **(E)** The positive rates of T-SPOT assay were compared by chi-square test.

PBMCs were isolated from TB patients, and the frequencies of LDGs were detected by FCM. Samples with high LDGs frequency (LDGs > 3%) were selected and divided into two parts, one of which were directly detected by T-SPOT assay. The number of mononuclear cells used for T-SPOT assay was adjusted to 1.5 × 10^5^/well according to the LDGs frequencies. LDGs in another part of PBMCs were removed. T-SPOT assay was then performed with the purified mononuclear cells at 1.5 × 10^5^ cells/well. Results showed that, after the removal of LDGs, the numbers of the ISCs significantly increased in response to both ESAT-6 (*P* = 0.0049) and CFP-10 (*P* = 0.0055) stimulation (Figure 3). The total ISCs in response to ESAT-6 and CFP-10 were also significantly increased in the condition of LDGs removal (*P* = 0.0041). In addition, the positive rate of T-SPOT assay increased from 77.78 to 94.44% after the removal of LDGs, but the difference was not significant (*P* = 0.1482) because the number of negative specimens was relatively small.

### Exogenous Addition of LDGs Decreased the ISCs and the Positive Rates of T-SPOT.TB Assay

**FIGURE 4:**
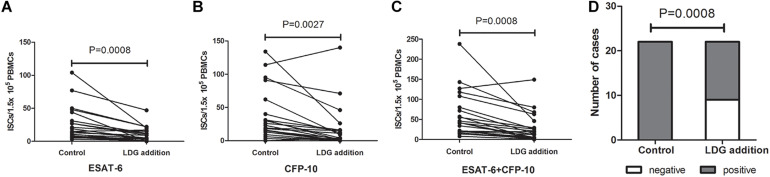
Addition of exogenous low-density granulocytes (LDGs) decreased the interferon-γ-secreting T cells (ISCs) in T-SPOT assay. Purified LDGs were added into the autologous peripheral blood mononuclear cells (PBMCs) of tuberculosis patients with low LDGs frequency (*n* = 18) to a final percentage of 11–30%. T-SPOT assays were performed with the untreated PBMCs and LDGs supplemented PBMCs, respectively, with the same numbers of mononuclear cells. The ISCs in response to **(A)** ESAT-6 and **(B)** CFP-10 stimulation and **(C)** the sum of the ISCs in response to ESAT-6 and CFP-10 were counted and compared by paired Student’s *t*-test. **(D)** The positive rates of T-SPOT assay were compared by chi-square test.

Peripheral blood from tuberculosis patients with low LDGs frequency (LDGs ≤ 3%) was infected with heat-killed *Mtb* at MOI of 5 (*Mtb* to neutrophils) for 2 h to induce the increase in LDGs. Then, the PBMCs in the infected peripheral blood were isolated, and LDGs in this PBMCs fraction were isolated by negative magnetic sorting. Purified LDGs with purity of above 90% were added into autologous PBMCs to a final percentage of 11–30%; then, these PBMCs with exogenous LDGs and another untreated autologous PBMCs were used to T-SPOT detection, with the same numbers of mononuclear cells. Results showed that, when compared to the untreated group, exogenous addition of LDGs significantly decrease the numbers of ISCs both in response to ESAT-6 (*P* = 0.0008) and CFP-10 (*P* = 0.0027) (Figure 4). The total ISCs in response to ESAT-6 and CFP-10 were also significantly decreased in the condition of LDGs addition (*P* = 0.0008). The positive rate of T-SPOT assay decreased from 100.00 to 59.09% after the addition of LDGs (*P* = 0.0008).

### The Frequencies of LDGs Correlate With the Production of IFN-γ in T Cells

**FIGURE 5:**
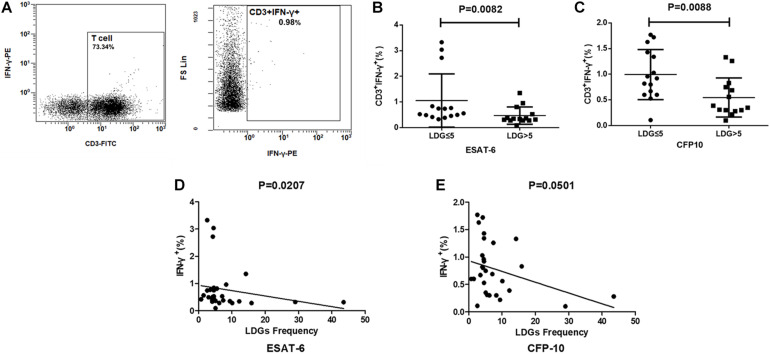
The frequencies of low-density granulocytes (LDGs) correlate with the production of interferon-gamma (IFN-γ) in T cells. **(A)** Representative flow-cytometry scatter plots of IFN-γ production in T cells. The production of IFN-γ in T cells response to **(B)** ESAT-6 and **(C)** CFP-10 in patients with high LDGs frequencies (*n* = 14) are significantly lower than those of low LDGs frequencies (*n* = 15). Spearman’s rho correlations between the LDGs frequency and the production of IFN-γ in T cells. **(D)** ESAT-6 (rho = -0.43; *P* = 0.0207). **(E)** CFP-10 (rho = -0.36; *P* = 0.0501).

T cell has been proven to be the main source of ISCs. To further clarify whether the effect of LDGs is achieved by targeting T cells, the production of IFN-γ in T cells was detected by FCM in tuberculosis patients. Results showed that the frequencies of IFN-γ-producing T cells were significantly lower in patients with high LDGs frequency than those with low LDGs frequency, both in response to ESAT-6 (*P* = 0.0088) and CFP-10 stimulation (*P* = 0.0082). Correlation analysis showed that, in response to ESAT-6 stimulation, the frequencies of IFN-γ-producing T cells were negatively correlated with the frequencies of LDGs (Figure 5).

### LDGs of Tuberculosis Patients Express High Level of PD-L1

**FIGURE 6:**
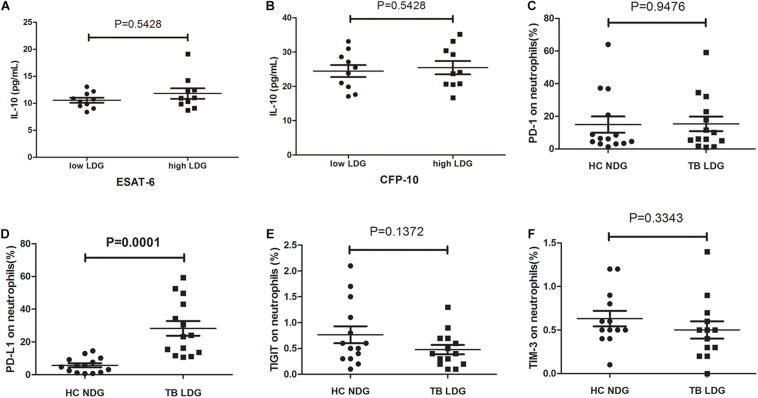
Secretion of interleukin (IL)-10 and expression levels of immunosuppressive costimulatory molecules in normal-density granulocytes (NDGs) of healthy controls and low-density granulocytes (LDGs) of tuberculosis patients. **(A,B)** Peripheral blood mononuclear cells (PBMCs) from tuberculosis patients with low (<3%, *n* = 10) or high (>10%, *n* = 10) percentages of LDGs were cultured for 24 h under the stimulation of *Mtb* antigens, and the IL-10 levels in culture supernatant were detected. **(C–F)** The frequencies of programmed death 1 (PD-1)-, programmed death ligand 1 (PD-L1)-, immunoreceptor tyrosine-based inhibitory (TIGIT)-, and T-cell immunoglobulin and mucin domain-containing protein 3 (TIM-3)-expressing LDGs in tuberculosis patients (*n* = 14), and the frequencies of PD- 1-, PD- L1-, TIGIT-, and TIM-3-expressing NDGs in healthy controls (*n* = 14) were determined and compared. The data were expressed as mean ± *SD*, and the statistical significance was determined by **(A,B)** unpaired Student’s *t*-test or **(C–F)** paired Student’s *t*-test.

The aforementioned results demonstrate that LDGs may exert suppressive effects on the T-cell responses to *Mtb* antigens and reduce the production of IFN-γ. Transcriptomic analysis in one of previous studies suggested that LDGs might secret IL-10 (La Manna et al., 2019), one of the well-known immunosuppressive cytokines. To investigate whether LDGs can suppress the IFN-γ production of T cells by secreting IL-10, we detected the IL-10 levels in culture supernatant of PBMCs with low (<3%) or high (>10%) percentages of LDGs. Results showed that there was no significant difference in the levels of IL-10 in the supernatant of PBMCs with different LDG ratios in response to both ESAT-6 and CFP-10 stimulation (Figure 6).

The immunosuppressive costimulatory molecules, such as programmed death 1 (PD-1), programmed death ligand 1 (PD-L1), T-cell immunoglobulin and mucin domain-containing protein 3 (TIM-3), and immunoreceptor tyrosine-based inhibitory (TIGIT) domains, were reported to play important roles in modulating the function of T cells (Singh et al., 2013, 2014; Wang et al., 2015; Jayaraman et al., 2016). Therefore, we compared the expression levels of PD-1, PD-L1, TIM-3, and TIGIT on NDGs and LDGs. Data showed that the frequency of PD-L1-positive cells in LDGs was significantly increased than that in NDGs (Figure 6, *P* = 0.0072). No significant difference was observed in the frequency of PD-1-expressing cells, TIM3-expressing cells, and TIGIT-expressing cells between LDGs and NDGs (Figure 6).

### Blocking PD-L1 Signal Significantly Increases the Number of ISCs

**FIGURE 7:**
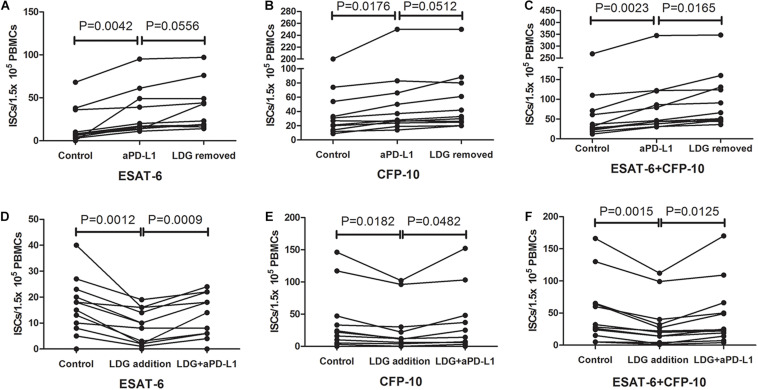
Blocking programmed death ligand 1 (PD-L1) signal increased the number of interferon-γ-secreting T cells (ISCs). Peripheral blood mononuclear cells (PBMCs) were collected from tuberculosis patients (*n* = 11) and treated with anti-PD-L1 monoclonal antibody or removal of low-density granulocytes (LDGs) by immunomagnetic bead method. These pretreated PBMCs were used together with untreated PBMCs for T-SPOT assay. The number of ISCs in response to **(A)** ESAT-6 and **(B)** CFP-10 and **(C)** the total number of ISCs in response to both ESAT-6 and CFP-10 were counted. LDGs from tuberculosis patients (*n* = 12) were isolated and treated with or without anti-PD-L1 monoclonal antibody, followed by adding into autologous freshly collected PBMCs. These PBMCs with exogenous LDG were used together with untreated PBMCs for T-SPOT assay. The number of ISCs in response to **(D)** ESAT-6 and **(E)** CFP-10 and **(F)** the total number of ISCs in response to both ESAT-6 and CFP-10 were counted. Statistical significance was determined by paired Student’s *t*-test.

PD-1/PD-L1 pathway has been reported to play an important role in subsiding immune responses. The observation that LDGs express a higher level of PD-L1 hinted us that LDGs might influence the T-SPOT assay through the PD-1–PD-L1 pathway. To verify this hypothesis, PBMCs were isolated from tuberculosis patients with high frequency of LDGs (>3%) and divided into three parts. One part was directly used for T-SPOT assay; the other part was treated with monoclonal antibody against PD-L1 (α-PD-L1, CD274 clone MIH1; eBioscience, 5 μg/ml) for 1 h, followed by removing the unbound antibodies by cell washing and T-SPOT assay, and the last part was detected by T-SPOT after removing the LDGs by immunomagnetic method. Results showed that, when compared to the untreated group, blockade of the PD-L1 by monoclonal antibody significantly increased the number of ISCs, both in response to ESAT-6 (*P* = 0.0042) and CFP-10 (*P* = 0.0176). The number of ISCs inα-PD-L1-treated group had no significant difference compared to LDG-removed group (Figures 7A,B). However, we found an increase in the total number of ISCs in LDG-removed group compared to the α-PD-L1-treated group (*P* = 0.0165, Figure 7C).

In order to further confirm that it is the PD-L1 molecules on LDGs that play a key role in this process, PD-L1 blockade was performed specifically on LDGs. Tuberculosis patients with low LDGs frequency (<3%) were selected. Peripheral bloods were collected from tuberculosis patients with low LDGs frequency (<3%) and infected with heat-killed *Mtb* to induce the increase in LDGs. PBMCs were isolated, and LDGs were purified by immunomagnetic negative sorting. Purified LDGs were treated with or without monoclonal antibody against PD-L1 (5 μg/ml) for 1 h. After removal of unbound antibodies, the same number of LDGs, treated, or untreated with PD-L1 antibody, were added into freshly collected autologous PBMCs to a final proportion of 10–20%. Finally, the mixture of PBMCs and exogenous LDGs was used to T-SPOT assay. Results showed that, when compare to the addition of untreated LDGs, the addition of PD-L1 antibody-treated LDGs significantly increased the number of ISCs in PBMCs, both in response to ESAT-6 (*P* = 0.002) and CFP-10 (*P* = 0.0025) (Figures 7D–F).

## Discussion

When the human body is infected with *Mtb*, the immune response will be activated, and specific immune effector cells and memory cells targeting *Mtb* will be primed. When exposed to the same antigen again, these memory cells will be activated rapidly and secrete large amount of IFN-γ. Therefore, if we stimulate the immune cells in the peripheral blood with *Mtb* antigens, we can judge whether the tested person is infected by *Mtb* by detecting the IFN-γ secreting T cells or the levels of IFN-γ. This technique is called IFN-γ release assay (IGRA). According to the different methods of specimen treatment and IFN-γ detection, IGRA can be divided into QuantiFERON gamma release assay and T-SPOT TB assay. For QuantiFERON gamma release assay, the whole blood was stimulated with *Mtb* antigens, and the levels of IFN-γ in plasma were detected by ELISA or chemiluminescence.

T-SPOT is a technique that uses the specific antigen of *Mtb* to stimulate and activate the *Mtb*-specific T cells in PBMCs, followed by detecting the amount of IFN-γ-secreting cells by ELISPOT method. In this study, a commercial T-SPOT detection kit was used. Two antigens that specifically expressed in *Mtb*, ESAT-6, and CFP-10, were used in this kit to activate the *Mtb*-specific T cells in PBMCs. A negative control without stimulation and a positive control with PHA stimulation were also set up. T-SPOT assay is currently the most sensitive, most commonly used technology to detect *Mtb* infection. However, it is susceptible to a variety of impact factors such as the immune status of patients and the activity, purity, and quantity of PBMCs. Previous studies have demonstrated that false negative result is one of the main problems encountered in T-SPOT assay (Nguyen et al., 2018; Li et al., 2019).

LDG is a population of polymorphonuclear granulocytes with lower density that locates in the PBMC layer after density gradient centrifugation. It has been found that the frequencies of LDGs were significantly increased in patients with various diseases, including tuberculosis, autoimmune diseases, and cancers (Balbi et al., 2018; Ho and Leung, 2018; Lee et al., 2018). Since patients with these diseases are the main population receiving T-SPOT assay, we want to know whether the LDGs mixed in PBMCs will affect the detection of T-SPOT. In this study, we first observed the relationship between the frequency of LDGs and the positive rate of T-SPOT assay in tuberculosis patients. The results showed that the positive rate of T-SPOT assay and the ISCs in patients with high frequency of LDGs was significantly lower than that in patients with low frequency of LDGs.

In order to explain this phenomenon, we first investigated whether LDGs itself secret IFN-γ in response to the stimulation of *Mtb* antigens. Our results showed that LDGs does not secrete IFN-γ whether or not they are stimulated by *Mtb* antigens. Therefore, the decrease in the relative number of T cells should be one of the factors affecting the results of T-SPOT assay in patients with high frequency of LDGs.

However, we found that the change in relative number of T cells cannot fully explain the decrease in *Mtb*-specific ISCs because the decrease in ISCs was significantly greater than that of T cells. Therefore, LDGs may have some other mechanisms to inhibit IFN-γ production in T cells. To confirm this speculation, the T-SPOT assays were performed under the conditions of removing LDGs in PBMCs and exogenously adding purified LDG in PBMCs, with the same number of mononuclear cells in each detection system. The results showed that after LDG removal, the number of ISCs in the same number of mononuclear cells increased significantly. The positive rate of T-SPOT assay also increased. However, the difference is not significant due to the relatively small number of T-SPOT negative specimens in tuberculosis patients. On the other hand, when purified LDGs were added into autologous PBMCs, the number of ISCs in the same number of PBMCs decreased significantly, and the positive rate of T-SPOT assay also decreased significantly. These results confirm that LDGs indeed can inhibit the secretion of IFN-γ in T cells through some mechanism. However, a previous study by Zhang et al. had reported an opposite impact of LDG removal on T-SPOT assay (Zhang et al., 2017). In this study, LDGs are removed by PBMCs adherence method and whole blood sedimentation method. However, considering that the adhesion ability of monocytes and B lymphocytes is stronger than that of granulocytes, these two methods remove not only LDGs but also monocytes and B lymphocytes, even some T cells. Therefore, the main reason for the inconsistency between these two studies may be due to the difference in cell removal methods.

The roles of LDG subset currently received much attention. However, the function of LDGs is inconsistent in different diseases. In some diseases such as antineutrophil cytoplasmic antibody-associated vasculitis and chronic granulomatous disease, they were described as proinflammatory cells (Denny et al., 2010; Grayson et al., 2015). However, they performed suppressive effects on T cells and were named as polymorphonuclear myeloid-derived suppressor cells (PMN-MDSCs) in some solid tumors and hematological malignancies (Sagiv et al., 2015; Moses and Brandau, 2016). Moreover, conflicting reports existed in the same disease regarding the function and role of LDGs. For instance, LDGs in SLE patients were named as PMN-MDSC by Wu et al. (2016) while considered to be proinflammatory cells by Rahman et al. (2019). Till now, the function of LDGs in tuberculosis remains to be elucidated.

In order to clarify the molecular mechanism of LDGs inhibiting the production of IFN-γ, we detected the levels of the main negative immune regulatory molecules including IL-10, TIGIT, TIM-3, PD-1, and PD-L1 in LDGs. The results showed that the expression level of PD-L1 on LDGs was significantly elevated. PD-L1 is the ligand of PD-1 and one of the main negative costimulatory molecules. PD-1 is mainly expressed on T cells and devotes to negatively regulate the activation and proliferation of T cells as an inhibitory molecule. In recent years, several studies had highlighted the critical roles of the PD-1/PD-L1 pathway in tuberculosis (Jurado et al., 2008; Alvarez et al., 2010; Singh et al., 2013, 2014, 2017). It was demonstrated that PD-1 was highly expressed on T cells in tuberculosis patients, and the levels of PD-1 on T cells was reported to correlate with the impaired functions of T cells and the severity of tuberculosis. Therefore, is it possible for LDG to inhibit the IFN-γ production and secretion in T cells through highly expressed PD-L1?

The results showed that the downregulation effect of LDGs on ISCs was significantly decreased when the PD-L1 signal was specifically blocked by anti-PD-L1 monoclonal antibody. These results demonstrated that LDGs can suppress the production of IFN-γ in T cells through PD-L1 signal. However, we also found an increase in the total number of ISCs in LDG-removed group compare to the α-PD-L1-treated group, which suggested that LDGs may also affect the IFN-γ production of T cells through some other mechanisms.

Our previous studies demonstrated that LDGs in tuberculosis patients are activated mature neutrophils that originated from *in situ* activation of peripheral neutrophils (Su et al., 2019), with elevated levels of membrane CD66b, CD15, and CD16. We demonstrate in this study that LDGs in tuberculosis patients can perform immunosuppressive roles on T cells. Consistent with our findings, a similar subset of mature neutrophils with CD66b^+^/CD11b^+^/CD16^+^ phenotype was also found to display powerful immunosuppressive activities in suppressing proliferation and IFN-γ production of T cells in patients with solid cancer (Lang et al., 2018).

In summary, our study shows that LDGs in tuberculosis patients can suppress the IFN-γ production of T cells and lead to the decrease in detection sensitivity of T-SPOT assay. Moreover, our study revealed that PD-L1 is highly expressed in LDGs and plays an important role in inhibiting the function of T cells. In addition, our study also showed that the positive rate of T-SPOT test could be improved by removing LDGs from PBMCs or treating PBMCs with anti-PD-L1 McAb before T-SPOT assay. This study suggests that negative T-SPOT results may not be reliable in patients with high frequency of LDGs. For patients with highly suspected *Mtb* infection, T-SPOT should be retested after removing LDGs from PBMCs or pretreating PBMCs with anti-PD-L1 McAb.

There is also a high frequency of LDGs in the peripheral blood of patients with HIV infection, cancer, and autoimmune diseases, and these patients are the main population receiving T-SPOT assay because of high risk of tuberculosis activation; whether LDGs in these patients also has similar function remains to be explored. To our knowledge, no previous study has investigated the role of LDGs in T-SPOT assay. Our study, for the first time, reveals that LDGs is one of the important factors regulating the production of IFN-γ in T cells and affecting the result of T-SPOT assay. Although more evidence is needed, our study also suggested for the first time that the LDGs in tuberculosis patients might function as PMN-MDSC in regulating the function of T cells.

## Data Availability Statement

The original contributions presented in the study are included in the article/supplementary material, further inquiries can be directed to the corresponding author/s.

## Author Contributions

JL and QL conceived and design the experiment. JR, RS, and YP performed the experiments. YG and ZH contributed significantly to acquisition of data, analysis, and interpretation of data. JR drafted the manuscript. YG, ZH, YY, YG, JL, and LZ helped perform the analysis with constructive discussions. All the authors have accepted responsibility for entire content of this submitted manuscript and approved submission.

## Conflict of Interest

The authors declare that the research was conducted in the absence of any commercial or financial relationships that could be construed as a potential conflict of interest.
